# The Use of Glycerine

**Published:** 1877-07

**Authors:** 


### SELECTIONS.

### THE USE OF GLYCERINE.

Several experiments on this subject, by M. Cattillon are reported in the London *Medical Record* :—

A notable diminution of the proportion of sugar was found in the blood of dogs submitted for long together to the influence of glycerine. But this influence of glycerine only seems to be exerted after ulta-therapeutic doses; and M. Cattillon was inclined to believe that the explanation of the favorable effects which glycerine may produce on diabetic patients must be sought in its action on the production of urea, and on the digestive functions. M. Cattillon has never found either sugar or albumen in the urine after the administration of glycerine in any dose whatsoever. Glycerine possesses decided laxative properties; a dose of 15 or 30 grammes induces, if taken into the stomach of an adult, an easy and soft motion; sometimes, too, the laxative effect does not increase with large doses administered all at once. Glycerine introduced into the stomach in large quantities, may act in two completely different ways, according as it is taken, in single large doses, or in repeated small doses. In the first case, when the proportion of 15 grammes to the kilogramme of the body-weight is reached, fatal accidents may supervene, and lesions comparable to those of acute alcoholism are found. In the second, on the contrary, no symptom but a rise in the temperature is manifested. It would appear that the rational dose of glycerine is from 15 to 30 grammes a day, if its functions for the restoration and regulation of the digestive functions are to be utilized. A dose of from 40 to 60 grammes, taken at once, may produce slight irritation of the kidneys and bladder. If we wish to give larger doses, as Dr. Harnach does in the treatment of diabetes (180 to 360 grammes, it is important to divide them so that they may be tolerated. These large doses do not appear to offer any advantage, and they bring on intestinal disturbances. They cause no other inconvenience; on the condition, however, that a quantity equivalent to 15 grammes to the kilogramme of the body-weight be not taken at once.—*Medical and Surgical Reporter.*

